# Infection Control Behavior at Home During the COVID-19 Pandemic: Observational Study of a Web-Based Behavioral Intervention (Germ Defence)

**DOI:** 10.2196/22197

**Published:** 2021-02-25

**Authors:** Ben Ainsworth, Sascha Miller, James Denison-Day, Beth Stuart, Julia Groot, Cathy Rice, Jennifer Bostock, Xiao-Yang Hu, Katherine Morton, Lauren Towler, Michael Moore, Merlin Willcox, Tim Chadborn, Natalie Gold, Richard Amlôt, Paul Little, Lucy Yardley

**Affiliations:** 1 Department of Psychology University of Bath Bath United Kingdom; 2 National Institute for Health Research Biomedical Research Centre Faculty of Medicine University of Southampton Southampton United Kingdom; 3 School of Psychology University of Southampton Southampton United Kingdom; 4 Primary Care Population Sciences and Medical Education University of Southampton Southampton United Kingdom; 5 Policy Research Unit London School of Hygiene & Tropical Medicine London United Kingdom; 6 Public Health England Behavioural Insights Public Health England London United Kingdom; 7 Centre for the Philosophy of Natural and Social Sciences London School of Economics London United Kingdom; 8 Behavioural Science Team Emergency Response Department Science and Technology Public Health England London United Kingdom; 9 School of Psychological Science University of Bristol Bristol United Kingdom

**Keywords:** COVID-19, novel coronavirus, behavior change, digital medicine, infection control, infectious disease, protection, digital health

## Abstract

**Background:**

To control the COVID-19 pandemic, people should adopt protective behaviors at home (self-isolation, social distancing, putting shopping and packages aside, wearing face coverings, cleaning and disinfecting, and handwashing). There is currently limited support to help individuals conduct these behaviors.

**Objective:**

This study aims to report current household infection control behaviors in the United Kingdom and examine how they might be improved.

**Methods:**

This was a pragmatic cross-sectional observational study of anonymous participant data from Germ Defence between May 6-24, 2020. Germ Defence is an open-access fully automated website providing behavioral advice for infection control within households. A total of 28,285 users sought advice from four website pathways based on household status (advice to protect themselves generally, to protect others if the user was showing symptoms, to protect themselves if household members were showing symptoms, and to protect a household member who is at high risk). Users reported current infection control behaviors within the home and intentions to change these behaviors.

**Results:**

Current behaviors varied across all infection control measures but were between *sometimes* (face covering: mean 1.61, SD 1.19; social distancing: mean 2.40, SD 1.22; isolating: mean 2.78, SD 1.29; putting packages and shopping aside: mean 2.75, SD 1.55) and *quite often* (cleaning and disinfecting: mean 3.17, SD 1.18), except for handwashing (*very often*: mean 4.00, SD 1.03). Behaviors were similar regardless of the website pathway used. After using Germ Defence, users recorded intentions to improve infection control behavior across all website pathways and for all behaviors (overall average infection control score mean difference 0.30, 95% CI 0.29-0.31).

**Conclusions:**

Self-reported infection control behaviors other than handwashing are lower than is optimal for infection prevention, although handwashing is much higher. Advice using behavior change techniques in Germ Defence led to intentions to improve these behaviors. Promoting Germ Defence within national and local public health and primary care guidance could reduce COVID-19 transmission.

## Introduction

The impacts of COVID-19 must primarily be tackled through changes in behavior undertaken by individuals and societies until a vaccine becomes available. In many countries (including the United Kingdom), people with COVID-19 infection are instructed to remain at home, together with cohabiting family or other household members, to prevent transmission between households. This increases the risk of within-household virus transmission. For example, in several environments where interhousehold movement is well controlled (eg, Taiwan, Ningbo, and Shenzen [1-3]), the virus continues to proliferate within close contacts.

To interrupt these transmission pathways, individuals must adopt *personal protective behaviors* [4]. Such targeted behaviors include handwashing, disinfection of surfaces, thorough cleaning and waste disposal, social distancing within the home (where possible), and wearing situationally appropriate personal protective equipment. A recent cohort study in Beijing, China demonstrated that performing these behaviors could dramatically reduce the likelihood of household transmission, but the highest risk of transmission was prior to symptom onset (typically before such behaviors are performed) [5]. Therefore, protective behaviors should be implemented before any household members develop symptoms. There is substantial individual variation in these behaviors, which are complex, environmentally and culturally dependent, and influenced by individual attitudes and beliefs [6]. Changing such complex behaviors effectively and rapidly within the context of COVID-19 requires an approach based on behavior change theory, evidence, and extensive participatory input [7].

Specific guidance for the public on protective behaviors has been developed in many countries and is widely recommended by politicians, the media, and public health and primary care networks [8]. However, few behavioral interventions have been used to support the public in these behaviors within their homes. A systematic review by our group has found evidence of only one digital intervention to date (Germ Defence [9,10]) that demonstrably improved health outcomes in respiratory tract infections within households. Germ Defence is a mobile-friendly website that provides targeted, tailored advice about how and why users should use infection control behaviors, aiming to supplement public health guidance with evidence- and theory-based behavior change techniques [11], optimized using extensive user feedback. In a large randomized controlled trial of 20,066 people (the PRIMIT [Primary Care Randomised Trial of an Internet Intervention to Modify Influenza-Like Illness and Respiratory Infection Transmission] trial) during the previous H1N1 (swine flu) pandemic [12], those randomized to use Germ Defence had reduced frequency and severity of respiratory tract infections, and reduced transmission to household members. Germ Defence is a freely available resource, and the intellectual property is held by the University of Southampton.

Germ Defence was rapidly adapted for the COVID-19 pandemic by a team of medical, public health and behavior change experts, and public contributors. It was then disseminated through multiple pathways (primarily but not exclusively in the United Kingdom), including public health and primary care networks (eg, by texting the website link to patients via general practitioner practices), national and local press, television coverage, and social media.

In this study, we aim to:

1. Examine current infection control behaviors in UK households
2. Compare current infection control behaviors with intentions to change behavior after using Germ Defence to control infection transmission

## Methods

### Design

This was a cross-sectional observational study of anonymous participant data from an active behavioral intervention. Consent was assumed from website use and acknowledged in the website privacy policy. All data was collected in line with General Data Protection Regulation EU Law. The study received ethical approval from the University of Bath (PREC reference 20-088). All time stamped data files used in analysis (and analysis scripts) are available at [13].

### Participants and Data

**Figure 1:**
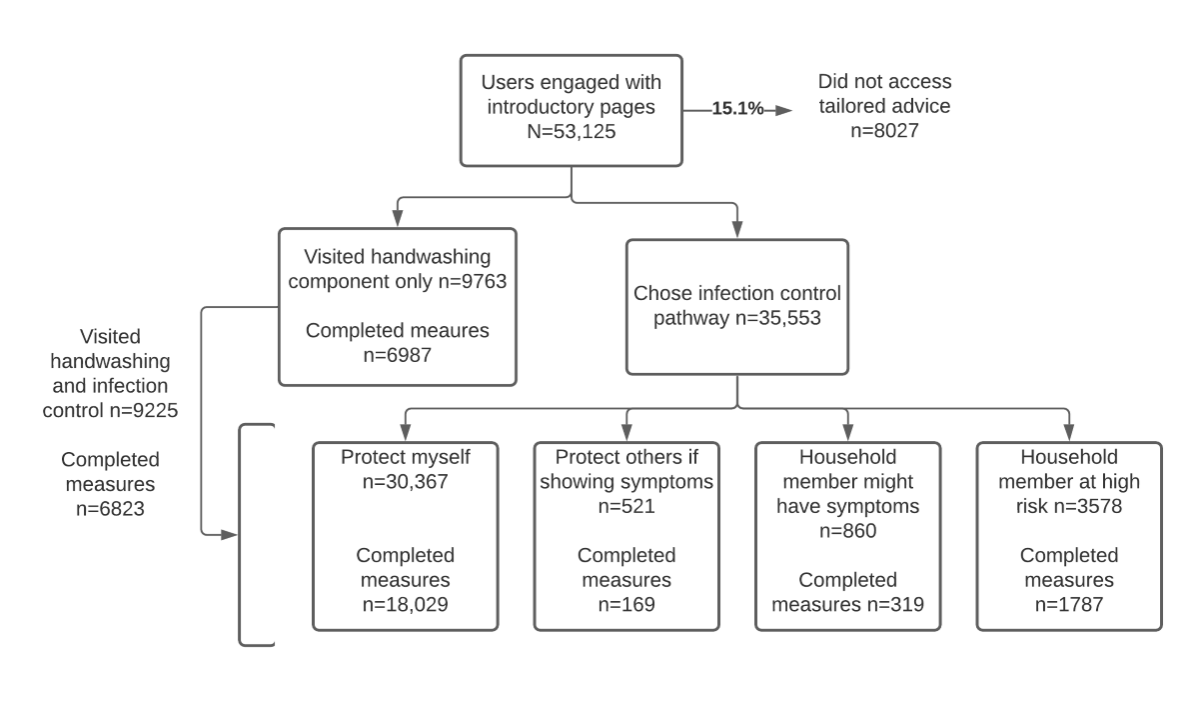
CONSORT (Consolidated Standards of Reporting Trials) diagram of Germ Defence website use and group categorization.

The data analyzed were collected from users of the Germ Defence website between May 6 and May 24, 2020. Usage was driven by media coverage, and users were encouraged to share the intervention on social media and by email. During this period, 70,566 website hits were recorded, with 53,125 users completing the introductory content (first 3 pages) and 28,285 people completing the core module, which included measures of current and intended behavior. Website use and engagement data was collected using Google Analytics embedded in the site (see Figure 1 for full CONSORT [Consolidated Standards of Reporting Trials] use diagram).

Data collection was kept to a minimum to reduce dropout. Behavioral measures were recorded through self-report questions within the website for current and intended behavior (see Table 1).

**Table 1:** Online self-report measures recorded during Germ Defence intervention

Behavior			Self-report item^a^
**Reducing illness infection control**			
	Social distancing	When you were/are with them, how often were you/do you plan to be more than 2 meters/6 feet away from the people you live with?	
	Cleaning/disinfecting	How often did you/do you plan to clean things that might have viruses on them?	
	Putting shopping/packages aside	How often did you/do you plan to put something aside for at least 1 day that might have viruses on it?	
	Self-isolating	How often did you/do you plan to spend time in a room on your own?	
	Wearing face coverings	How often did you/do you plan to wear a face covering and glasses (and safely remove and clean them) when you are in the same room as other people?	
**Handwashing behavior**			
	Before snacking	How often did you/do you plan to wash your hands before you ate/eat with your fingers (eg, snack, fruit, or sweets)?	
	After coming home	How often did you/do you plan to wash your hands when you came/come into a house (eg, after work, shopping, travelling)?	
	After coughing	How often did you/do you plan to wash your hands after blowing your nose or sneezing/coughing on your hands?	
	After coming into contact with possible carrier	How often did you/do you plan to wash your hands after you had been/being close to someone who may have a virus (within 6 feet)?	
	After touching something	How often did you/do you plan to wash your hands after touching something that lots of other people have touched (eg, doors, money, or handrails)?	
**Website helpfulness (recorded on a scale of 1-10)**			
	Helpfulness score	How strongly do you agree or disagree that Germ Defence was helpful to you?	

^a^Measures were all scored on a Likert scale with answers of 1 (almost never), 2 (sometimes), 3 (quite often), 4 (very often), and 5 (almost always). Users could also answer *not applicable* (eg, if they lived alone and therefore did not need to socially isolate within their household).

### Intervention

Germ Defence content was developed using theoretical modeling and qualitative research [14] in line with the person-based approach [15], drawing principally on the theory of planned behavior [16], Leventhal’s common sense model of illness [17], and protection motivation theory [18]. The intervention content, design, and structure were optimized iteratively using in-depth qualitative think-aloud interviews with public contributors (authors JB and CR) and members of the public to ensure the intervention was accessible, credible, and motivating for as many people as possible [15].

Based on process evaluations of the original randomized controlled trial [12] and a previous public dissemination [19], Germ Defence has been updated and streamlined for use during the COVID-19 outbreak, including broadening the infection control behaviors that were recommended. The intervention is a single session designed to be easily accessible with no sign-up or password required. Full details of the intervention structure and development are reported elsewhere [3,16,18,19] and archived copies are available at [20] (see Germ Defence v3). Intervention content was “frozen” during the reported data collection period. A structured outline of content is available in Textbox 1.

**A detailed outline of Germ Defence content and structure (note: the website and all associated content can be accessed for free).**

**Introductory content (3 pages)**

- Introductory pages seek to increase users’ perceived risk by emphasizing the personal and social health consequences of contracting COVID-19. These are followed by messages to increase skills and confidence to reduce exposure to the virus.

**Website pathway selection (2 pages)**

- To allow users to choose the advice they consider most personally relevant, the intervention is structured so that users initially select between two components of interest: handwashing and reducing Illness. The reducing illness component is tailored such that a user selects one of four streams of content (each lasting 11 pages) that is relevant to the user’s situation: (1) to protect themselves generally, (2) to protect others if the user was showing symptoms, (3) to protect themselves if household member(s) showed symptoms, or (4) to protect a household member who is at high risk. The advice is tailored in this way to encourage users to adopt behaviors appropriate to the perceived level and pattern of risk in their household. For example, users in the protect themselves generally group would vary from very low to very high risk. It was not possible to provide specific tailored advice for every household combination of risks and resources (eg, based on the need and potential for household members to self-isolate within the home); therefore, Germ Defence aimed to educate users to adopt behaviors that were appropriate and feasible for their own circumstances.

**Tailored infection control behavior advice (7 pages)**

- Clear and detailed advice is then provided for self-isolating, social distancing, disinfecting/cleaning, wearing face coverings, and putting items aside that may have viruses on them such as shopping/packages. Advice is provided to the extent that users feel is appropriate for the perceived risk. These pages also contain ideas and information on how to structure the home and engage in behaviors safely. The handwashing component provides advice focused on handwashing that is relevant to all groups over 5 pages.

**Goal-setting advice (3 pages)**

- Both the handwashing and reducing illness components contain goal-setting sections where users indicate their behavior over the past week, view a motivational message, and then plan their behavior for the future. Users who do not select any improvement are encouraged to review their plan. After completing either the handwashing or reducing illness components, users are asked how helpful they found the website.

**Additional information**

- Users are then able to revisit the first two components, choose from two additional components with more detailed information about the same behaviors (eg, how to social distance with young children, how to stop touching your face), or view details about the website.

### Statistical Analysis

We included data from all users who accessed the website during the study period.

For analysis, users were grouped according to the tailored website pathway they selected within the *reducing illness* component (*protect myself generally* vs *protect others if I am showing symptoms* vs *protect myself if a household member has symptoms* vs *protect a household member at high risk*). Users could also view the handwashing component, which was relevant to all groups. If they did not view *reducing illness*, they were not included in group comparisons, but handwashing responses were still recorded. Users could complete more than one type of tailored pathway, but we only analyzed responses for the pathway that was selected first.

To understand current infection control behaviors (aim 1), behavioral measures were analyzed individually and collapsed together to form an *average infection control behavior* score. When users completed a plan more than once (eg, if they received website feedback that their initial plan could be further improved), the *final* plan was used. If users did not think a behavior was relevant to them (eg, they lived alone so did not need to socially isolate or could not socially isolate from young children), they could answer *not applicable*. This was coded as missing data and not included in analysis. Linear regression compared between-group scores for behavior.

To compare current behaviors with intended behavior after using Germ Defence (aim 2), linear regression models comparing between-group scores for intentions controlled for current behavior were used. Paired *t* test comparisons examined the difference between current behavior and intended behavior within groups.

## Results

### Use of the Germ Defence Website

We considered data from 53,125 users who completed at least the initial introductory website pages. Users accessed Germ Defence from 129 countries (a full CONSORT diagram of use is presented in Figure 1). The majority (n=44,446, 83.7%) of users were from the United Kingdom (England: n=40,164, 75.6%; Scotland: n=2204, 4.2%; Wales: n=1459, 2.8%; Northern Ireland: n=566, 1.1%; other: n=73, 0.1%). The mean use time was 8 minutes 28 seconds, and the mean number of pages viewed was 19.9. Of the recorded sessions, 54.1% (n=28,740) lasted longer than 1 minute. Over half (n=28,687, 54%) of the users accessed Germ Defence using a mobile device, 31% (n=16,469) accessed with a tablet, and 15% (n=7968) with a desktop or laptop computer. Only 10.6% (n=5631) of users were *return users* visiting for a second time. Aggregated use statistics for users outside the United Kingdom are provided in Multimedia Appendix 1. Detailed use for each website component is presented in Figure 1. The overall mean helpfulness of the website was rated as 7.77 (SD 2.31) out of 10.

### Infection Control Behaviors and Intended Behaviors in Users of Germ Defence

All groups (protect themselves generally, protect others if the user was showing symptoms, protect themselves if household members were showing symptoms, and protect a household member who is high risk) reported using most current infection behaviors sometimes or quite often within the home. Overall, users reported they would wear a face covering almost never or sometimes (mean 1.61, SD 1.19) and would socially distance sometimes or quite often (mean 2.40, SD 1.22). Users reported socially isolating in their own room sometimes or quite often (mean 2.78, SD 1.29) and putting packages and shopping aside sometimes or quite often (mean 2.75, SD 1.55). Users reported cleaning and disinfecting quite often or very often (mean 3.17, SD 1.18).

Frequency of the five infection control behaviors from the *reducing illness* pathway within each group is reported in Table 2 (with handwashing reported in a separate table), as well as mean differences and 95% CIs of group comparisons (each group vs the *protect themselves generally* group). The frequency of behaviors did not vary appreciably between groups; numerically, the *protect themselves generally* group were least likely to socially distance (mean 2.39, SD 1.22). People in the *protect others if user showing symptoms* group were least likely to clean and disinfect (mean 2.95, SD 1.26) and put aside shopping and packages (mean 2.39, SD 1.48) but most likely to wear a face covering (mean 1.91, SD 1.36). People in the *protect themselves if household members showing symptoms* group were most likely to maintain social distance (mean 2.57, SD 1.23), and users in the *protect household members at high risk* group were least likely to stay in their own room (mean 2.64, SD 1.16) and least likely to wear a face covering (mean 1.42, SD 0.99).

Table 2 shows some small differences in how often participants planned to perform behaviors in the future (corrected for levels of current behavior) between groups. Compared to people in the protect themselves generally group, people showing symptoms planned to clean and disinfect, and put aside shopping less frequently, but they planned to self-isolate more frequently. People in the protect themselves from household member with symptoms group planned to socially distance and self-isolate more frequently than those in the protect themselves generally group. People looking to protect a high-risk household member planned to conduct all of the behaviors slightly more frequently than the protect themselves generally group.

Paired *t* test comparisons examined differences between current and planned behaviors after using the Germ Defence website. Mean difference scores for each group and 95% CIs are reported in Table 3. The difference between intended and current behavior was largest for cleaning and disinfecting (mean difference 0.38, 95% CI 0.37-0.39) and putting aside shopping and packages (mean difference 0.49, 95% CI 0.47-0.50), and was lowest for self-isolating (mean difference 0.15, 95% CI 0.14-0.16). Overall, infection control behaviors increased (mean difference 0.30, 95% CI 0.29-0.31).

Handwashing behavior is reported in Table 4. Mean current handwashing behavior was higher than other infection control behaviors (mean 4.04, SD 0.84) with reported intended behavior consistently higher (mean increase 0.41, 95% CI 0.40-0.42).

**Table 2:** Current and intended infection control behaviors.

Behaviors		Protect themselves generally (n=18,029)^a^, mean (SD)	Protect others if user showing symptoms (n=169)			Protect themselves if household member showing symptoms (n=319)			Protect a household member at high risk (n=1787)			
			Mean (SD)	Mean difference (95% CI)	Cohen *d*^b^	Mean (SD)	Mean difference (95% CI)	Cohen *d*		Mean (SD)	Mean difference (95% CI)	Cohen *d*
**Current behavior**												
	Social distancing	2.39 (1.22)	2.52 (1.39)	0.13 (–0.07 to 0.33)	0.11	2.57 (1.23)	0.17 (0.04 to 0.31)	0.15		2.51 (1.20)	0.12 (0.06 to 0.18)	0.10
	Clean/disinfect	3.18 (1.18)	2.95 (1.26)	–0.24 (–0.42 to –0.06)	0.20	3.05 (1.18)	0.17 (0.04 to 0.31)	0.11		3.19 (1.17)	0.003 (–0.05 to 0.06)	0.00
	Put aside shopping/packages	2.74 (1.55)	2.39 (1.48)	–0.35 (–0.60 to –0.11)	0.23	3.00 (1.49)	0.26 (0.08 to 0.44)	0.17		2.82 (1.59)	0.08 (0.004 to 0.16)	0.05
	Self-isolate in own room	2.79 (1.30)	2.85 (1.43)	0.05 (–0.15 to 0.25)	0.04	2.75 (1.26)	–0.04 (–0.19 to 0.10)	0.03		2.64 (1.16)	–0.15 (–0.21 to –0.08)	0.11
	Wear face covering	1.63 (1.21)	1.91 (1.36)	0.28 (0.07 to 0.49)	0.24	1.75 (1.28)	0.12 (–0.02 to 0.27)	0.10		1.42 (0.99)	–0.21 (–0.27 to –0.14)	0.17
	Overall behavior score^c^	2.67 (0.91)	2.61 (1.08)	–0.05 (–0.19 to 0.08)	0.06	2.68 (0.90)	0.01 (–0.09 to 0.11)	0.01		2.59 (0.80)	–0.07 (–0.12 to –0.03)	0.08
**Intended Behavior**												
	Social distancing	2.63 (1.28)	2.79 (1.47)	0.05 (–0.06 to 0.16)^d^	0.12	2.88 (1.30)	0.12 (0.05 to 0.20)^d^	0.19		2.84 (1.27)	0.11 (0.07 to 0.14)^d^	0.16
	Clean/disinfect	3.57 (1.16)	3.18 (1.33)	–0.14 (–0.25 to –0.03)^d^	0.33	3.46 (1.18)	0.001 (–0.08 to 0.08)^d^	0.09		3.63 (1.15)	0.05 (0.01 to 0.08)^d^	0.05
	Put aside shopping/packages	3.24 (1.52)	2.73 (1.59)	–0.19 (–0.34 to –0.04)^d^	0.34	3.44 (1.41)	–0.02 (–0.12 to 0.09)^d^	0.13		3.37 (1.52)	0.06 (0.01 to 0.11)^d^	0.08
	Self-isolate in own room	2.94 (1.28)	3.08 (1.41)	0.10 (0.02 to 0.18)^d^	0.12	2.97 (1.23)	0.07 (0.01 to 0.13)^d^	0.03		2.87 (1.17)	0.06 (0.04 to 0.09)^d^	0.05
	Wear face covering	1.95 (1.37)	2.19 (1.50)	0.03 (–0.11 to 0.17)^d^	0.18	2.15 (1.47)	0.08 (–0.01 to 0.18)^d^	0.15		1.82 (1.28)	0.08 (0.03 to 0.12)^d^	0.09
	Overall behavior score	2.97 (0.96)	2.86 (1.20)	–0.03 (–0.12 to 0.05)^d^	0.11	3.01 (0.96)	0.03 (–0.03 to 0.09)^d^	0.04		2.97 (0.89)	0.06 (0.03 to 0.08)^d^	0.00

^a^Between group comparisons compare each group to the protect themselves generally group. Scale: 1 is almost never, 2 is sometimes, 3 is quite often, 4 is very often, and 5 is almost always.

^b^Reported as the standardized mean difference between each group and the comparison group.

^c^Overall behavior scores are means calculated from all behaviors in which a response was recorded.

^d^Controlling for current behavior.

**Table 3:** Group differences between behavior and intention.

Behaviors		Protect themselves generally (n=18,029)^a^		Protect others if user showing symptoms (n=169)		Protect themselves if household member showing symptoms (n=319)		Protect a household member at high risk (n=1787)		Overall	
		Mean difference (95% CI)	Cohen *d*	Mean difference (95% CI)	Cohen *d*	Mean difference (95% CI)	Cohen *d*	Mean difference (95% CI)	Cohen *d*	Mean difference (95% CI)	Cohen *d*
**Behavior**											
	Social distancing	0.22 (0.21-0.23)	0.35	0.26 (0.11-0.40)	0.30	0.33 (0.24-0.42)	0.41	0.31 (0.28-0.35)	0.43	0.23 (0.22-0.24)	0.36
	Clean/disinfect	0.38 (0.37-0.39)	0.52	0.30 (0.17-0.44)	0.36	0.41 (0.31-0.51)	0.47	0.43 (0.39-0.47)	0.54	0.38 (0.37-0.40)	0.52
	Put aside shopping/packages	0.49 (0.47-0.50)	0.49	0.39 (0.24-0.54)	0.42	0.41 (0.31-0.51)	0.47	0.53 (0.48-0.58)	0.50	0.49 (0.47-0.50)	0.49
	Self-isolate in own room	0.14 (0.13-0.15)	0.28	0.23 (0.11-0.36)	0.30	0.21 (0.14-0.29)	0.33	0.22 (0.19-0.25)	0.34	0.15 (0.14-0.16)	0.29
	Wear face covering	0.28 (0.27-0.30)	0.37	0.29 (0.12-0.47)	0.30	0.35 (0.25-0.46)	0.42	0.37 (0.33-0.42)	0.42	0.29 (0.28-0.29)	0.37
Average infection control score		0.29 (0.29-0.30)	0.53	0.27 (0.16-0.38)	0.38	0.32 (0.25-0.40)	0.49	0.36 (0.33-0.39)	0.57	0.30 (0.29-0.31)	0.53

^a^Group n values are taken across all behaviors.

**Table 4:** Paired comparisons between current and intended handwashing behavior.

Handwashing situation	Current behavior (n=12,981), mean (SD)	Intended behavior (n=12,981), mean (SD)	Mean difference (95% CI)	Cohen *d*
Before eating snacks	3.91 (1.28)	4.45 (0.99)	0.54 (0.52-0.56)	0.54
After coming home	4.66 (0.81)	4.80 (0.62)	0.14 (0.13-0.15)	0.26
After sneezing or coughing	3.45 (1.43)	4.11 (1.23)	0.66 (0.64-0.68)	0.59
After contact with possible carrier	4.22 (1.24)	4.53 (1.00)	0.30 (0.29-0.32)	0.36
After touching something	4.13 (1.23)	4.50 (0.97)	0.36 (0.35-0.38)	0.43
Overall score^a^	4.00 (1.03)	4.34 (0.91)	0.34 (0.33-0.35)	0.50

^a^Handwashing overall score was a separate item

## Discussion

### Summary of Findings

Germ Defence was accessed by a large number of users across 129 countries, primarily from the United Kingdom. This demonstrates public interest in adopting appropriate infection control behaviors in the home during the COVID-19 pandemic. After using Germ Defence, all groups reported intentions to increase the frequency of their infection control behaviors, including handwashing.

Except for handwashing, self-reported infection control behaviors in the home were only reported *sometimes or quite often* regardless of whether people were seeking to protect themselves, concerned about demonstrating COVID-19 symptoms, had a household member showing symptoms, or were seeking to protect a high-risk household member. The frequency of wearing face coverings was consistently the lowest of the behaviors, while cleaning and disinfecting was the most frequently reported of the behaviors outside of handwashing. All of these infection control behaviors were reported to be performed much less frequently than handwashing.

As would be expected, certain behaviors and intentions varied according to the circumstances of groups; for example, people seeking to protect others when showing symptoms reported higher current frequencies of wearing face coverings, while people seeking to protect a high-risk household member reported the intention to socially distance within the home more frequently.

### Comparison With Existing Literature

This study provides the first up-to-date analysis of infection control behaviors and intentions across the United Kingdom in a large sample during the COVID-19 pandemic. Within-household transmission will be increasingly important as infection control measures become established in external, public environments [6,21]. Therefore, understanding current infection control behaviors within homes (and how to improve them) is vital to continue controlling the pandemic.

Self-reported infection control behaviors other than handwashing are lower than is optimal for infection prevention, even in Germ Defence users who were likely more motivated and willing to engage in protective behaviors than the general population (as they were seeking additional information) [22]. Increasing engagement in these behaviors is important as societal restrictions are released and perceived risk reduces [23].

Germ Defence users reported intentions to increase the frequency of infection control behaviors over their current rates. Although such intentions potentially misrepresent the observed behavioral change after an intervention (the *intention-behavior gap* [24]), our evidence suggests that Germ Defence may overcome this. Analysis of comparable data from the PRIMIT trial handwashing intervention showed slightly smaller behavior and intention differences (Cohen *d*=0.45). This change was sufficient to cause reduced infection transmission and severity within households after 16 weeks [12]. Comparable data during the current pandemic (reducing illness behaviors: Cohen *d*=0.53; handwashing: Cohen *d*=0.50) shows a slightly larger effect across a broader range of behaviors that may have a larger impact on infection rates.

### Study Limitations

As a cross-sectional observation of an active intervention, Germ Defence lacks longitudinal follow-up. Care must be taken when interpreting findings within the rapidly changing context of the COVID-19 pandemic. Our method of categorization using website pathways may not be accurate for some users or might overlook individual differences within categories.

Our data may not be a representative sample from the wider UK population for several reasons. First, users of Germ Defence are likely to be more motivated and report higher frequencies of infection control behaviors. Second, although analytic data indicates that the large majority of the intervention’s users were from the United Kingdom, we could not identify non-UK users within behavioral data. Finally, self-reported infection control behaviors may not be accurate reflections of actual behaviors occurring within households.

However, none of these limitations affect our main findings; indeed, people are prone to overreport protective behaviors, further highlighting the need for improvement.

### Implications for Practice and Research

A concerted effort to improve household infection control behaviors across the UK population is likely to be an efficient use of health resources, both to reduce current rates of infection and to prevent the likelihood and severity of future outbreaks. Handwashing behaviors are already relatively high—perhaps due to existing familiarity with the behavior supported by a focus in public health advice on increasing handwashing in earlier stages of the pandemic. Therefore recommending digital interventions such as Germ Defence to target other infection control behaviors within the home may help control the current pandemic.

Given the current rates of infection control behaviors within the home even within a motivated sample, it is vital to address barriers to engaging in them. For example, people living in crowded, working households are more likely to come into contact with the virus [5] and may find it difficult to self-isolate. Similarly, cultural differences, financial challenges, or caring responsibilities may cause barriers to social distancing [6]. Research should explore how to support these behaviors for as many households as possible. Indeed, digital interventions such as Germ Defence can use tailored content to target behaviors that are relevant for specific user groups.

### Conclusion

Our findings show substantial room for improvement in protective behaviors across the United Kingdom—even in our motivated, self-selected sample—as societal restrictions are eased. People are not sufficiently self-isolating within the home to prevent household transmission, even when a household member or the individual themselves are demonstrating COVID-19 symptoms. Promoting evidence-based behavior change interventions might improve these behaviors, reducing transmission within households and the incidence and severity of infections.

Germ Defence is a scalable, evidence-based, acceptable, and free public health intervention with negligible safety risk, which could be included in public heath guidance and promoted via primary care networks at minimal cost for wide population coverage.

## Appendix

Multimedia Appendix 1 Comparison of aggregated use statistics for users outside of the United Kingdom (compared to users within the United Kingdom).

## Abbreviations

CONSORT: Consolidated Standards of Reporting Trials
NIHR: National Institute for Health Research
PRIMIT: Primary Care Randomised Trial of an Internet Intervention to Modify Influenza-Like Illness and Respiratory Infection Transmission
UKRI: United Kingdom Research and Innovation
