# Supplementary material for: Infection Control Behavior at Home During the COVID-19 Pandemic: Observational Study of a Web-Based Behavioral Intervention (Germ Defence)
Source: J Med Internet Res. 2021 Feb 25;23(2):e22197. doi: 10.2196/22197 (PMC7909304; doi:10.2196/22197)
Supplement: Multimedia Appendix 1 [file jmir_v23i2e22197_app1.docx]

**Appendix 1**

Comparison of aggregated usage statistics for users outside of the UK (compared to users within the UK).

|  | **UK** | **Non-UK** |
| --- | --- | --- |
| **Unique users (N)** | 42,316 | 8,320 |
| **Total sessions (N)** | 43,943 | 8,525 |
| **Returning Visitors (%)** | 11.3% | 9.3% |
| **Mobile Users (%)** | 53.5% | 56.8% |
| **Average Session Duration** | 08:43 | 07:13 |
| **Average pages visited per session** | 19.46 | 16.08 |

**Note:** Data was collected using Google Analytics. No geographic data available for 2,489 users due to privacy settings.
